# Comparative proteomics study of proteins involved in induction of higher rates of cell death in mitoxantrone-resistant breast cancer cells MCF-7/MX exposed to TNF-α

**DOI:** 10.22038/ijbms.2020.40029.9486

**Published:** 2020-05

**Authors:** Saeed Norouzi, Rezvan Yazdian Robati, Morteza Ghandadi, Khalil Abnous, Javad Behravan, Fatemeh Mosaffa

**Affiliations:** 1 Department of Pharmaceutical Biotechnology, School of Pharmacy, Mashhad University of Medical Sciences, Mashhad, Iran; 2 Molecular and Cell Biology Research Center, Faculty of Medicine, Mazandaran University of Medical Sciences, Sari, Iran; 3 Pharmaceutical Sciences Research Center, Mazandaran University of Medical Sciences, Sari, Iran; 4 Department of Pharmacognosy and Pharmaceutical Biotechnology, Faculty of Pharmacy, Mazandaran University of Medical Sciences, Sari, Iran; 5 Pharmaceutical Research Center, Pharmaceutical Technology Institute, Mashhad University of Medical Sciences, Mashhad, Iran; 6 School of Pharmacy, University of Waterloo, Waterloo, Ontario, Canada; 7 Biotechnology Research Center, Pharmaceutical Technology Institute, Mashhad University of Medical Sciences, Mashhad, Iran

**Keywords:** 14-3-3 γ, MCF-7/MX cells, Mitoxantrone, Multidrug resistance, TNF-α

## Abstract

**Objective(s):**

Resistance to medications is one of the main complications in chemotherapy of cancer. It has been shown that some multidrug resistant cancer cells indicate more sensitivity against cytotoxic effects of TNF-α compared to their parental cells. Our previous findings indicated vulnerability of the mitoxantrone-resistant breast cancer cells MCF-7/MX to cell death induced by TNF-α compared to the parent cells MCF-7. In this study, we performed a comparative proteomics analysis for identification of proteins involved in induction of higher susceptibility of MCF-7/MX cells to cytotoxic effect of TNF-α.

**Materials and Methods:**

Intensity of protein spots in 2D gel electrophoresis profiles of MCF-7 and MCF-7/MX cells were compared with Image Master Platinum 6.0 software. Selected differential protein-spots were identified with MALDI-TOF/TOF mass spectrometry and database searching. Pathway analyses of identified proteins were performed using PANTHER, KEGG PATHWAY, Gene MANIA and STRING databases. Western blot was performed for confirmation of the proteomics results.

**Results:**

Our results indicated that 48 hr exposure to TNF-α induced 87% death in MCF-7/MX cells compared to 19% death in MCF-7 cells. Forty landmarks per 2D gel electrophoresis were matched by Image Master Software. Six proteins were identified with mass spectrometry. Western blot showed that 14-3-3γ and p53 proteins were expressed higher in MCF-7/MX cells treated with TNF-α compared to MCF-7 cells treated with TNF-α.

**Conclusion:**

Our results showed that 14-3-3 γ, prohibitin, peroxiredoxin 2 and P53 proteins which were expressed differentially in MCF-7/MX cells treated with TNF-α may involve in the induction of higher rates of cell death in these cells compared to TNF-α-treated MCF-7 cells.

## Introduction

Despite all the advantages of chemotherapy in overcoming cancer, incidence of multidrug resistance (MDR) phenotype in most cases of cancer is high (1). Prolonged exposure to chemotherapeutic agents leads to decreased sensitivity of cancer cells to drugs and development of MDR. MDR is the main cause of failure of approximately 90 % of cancer therapy cases (2-5). Recently scientists concentrate on invention of new therapeutic methods including immuno-therapeutic methods, cancer vaccines, stimuli-responsive nanomedicine and designing novel delivery strategies to improve site-specific accumulation of drugs leading to elevation of chemotherapeutic protocols efficiency and attenuation of drug resistance development in cancer cells (6-10). 

The most relevant mechanism in converting a chemo- sensitive cancer cell to its chemo-resistant form is up-regulation of proteins enabling cancer cells to efflux chemo-drugs out of the cell. These proteins including P-glycoprotein (P-gp), breast cancer resistance protein (BCRP, ABCG2) and multidrug resistance proteins (MRPs) belong to ATP-binding caste (ABC) transporters (11). In addition, the elevation of DNA repair system efficiency, changing in drug metabolism processes and alteration in signal transduction pathways are other events that may participate in promoting survival and self-renewal capacity of cancer cells undergoing chemotherapy protocols. Based on these mechanisms, it seems that drug resistant cancer cells exhibit more vulnerability to some external stimuli compared to their chemo sensitive parental cells (2, 12, 13). In several studies, a new phenomenon named collateral sensitivity (CS) has been observed. This phenomenon is witnessed in tumors overexpressing ABC family of transporters such as P-gp, MRP1/ABCC1 and BCRP/ABCG2 and is one of the promising procedures to treat drug resistant cancer cells (14, 15). CS is the ability of compounds killing MDR cells selectively higher than their parental cells (4). Some studies have revealed that chemotherapy drug resistant cancer cells such as CEM/V (vinblastin resistant leukemia), 8226/D (doxorubicin resistant myeloma), FLC-DXR (doxorubicin resistant friend erythroleukemia cell line) and B16-DXR (doxorubicin resistant melanoma) are more sensitive to cytotoxic effects of TNF-α compared to their parental cell lines (16-20).

Tumor necrosis factor-α (TNF-α) belongs to pro-inflammatory cytokines and exerts pleiotropic effects on many different cellular processes. TNF-α signal transduction through its specific receptors TNF-RI and TNF-RII activates many signaling factors such as NF-kB, c-jun N-terminal kinase (JNK), caspases, phosphatidylinositide 3-kinase (PI3K)/Akt and leads to the production of reactive oxygen species (ROS) (21).

According to our previous studies, TNF-α exerts more cytotoxic effects in MCF-7/MX cells through caspase independent pathways by activation of receptor-interacting serine/threonine-protein kinase 1 (RIPK1) and increased accumulation of intracellular reactive oxygen species (ROS). On the other hand, activation of NF-kB, elevated levels of antioxidant enzymes and increased levels of phosphorylated AKT are involved in the resistance of MCF-7 cells to TNF-α cytotoxic effects (21, 22).

We designed a comparative proteomics study between proteome profiles of both MCF-7/MX and MCF-7 cell lines following exposure to TNF-α. The main purpose of this investigation was to expand our findings about proteins and signal transduction pathways contributing to higher cytotoxic effects of TNF-α on MCF-7/MX cells in comparison with MCF-7 cells. The comparative studies between cancer cells and their drug resistant forms provides information that may be used to design new anti-cancer drugs and therapeutic regimes and finding more compounds with CS ability in the future.

## Materials and Methods

***Reagents***

Iodoacetamide and 3-[(3-cholamidopropyl) dimethylammonio]-1-propanesulfonate hydrate (CHAPS), and were purchased from Sigma Aldrich, USA. Dithiothreitol (DTT), urea, sodium dodecyl sulfate (SDS) and thiourea were prepared from Merck, Germany. Ampholyte with pH 3–10, IPG Strips length 17 cm with pH 3–10 nonlinear and protease inhibitor cocktail were purchased from Bio Rad, USA. All primary antibodies including β-actin, 14-3-3 ɣ and P53 were bought from Cell Signaling Technology, USA. Fetal bovine serum (FBS), RPMI 1640 medium, and penicillin were prepared from GIBCO, USA. Apoptosis detection kit was bought from Sigma-Aldrich, USA. Both cell lines, MCF-7 and MCF-7/MX were received as a gift from Dr. Erasmus Schneider, Wadsworth Center, New York State Department of Health, USA.

***Cell culture***

Both MCF-7 and MCF-7/MX cells were seeded in T25 flasks in RPMI 1640 culture medium with 10% fetal bovine serum. The seeded cells were incubated at 37 ^°^C in the presence of 5 % CO_2_. MCF-7/MX cells were grown on RPMI 1640 medium supplemented with 100 nM mitoxantrone for 100 hr in incubator for elevation of their resistance to mitoxantrone.

***Phase contrast imaging***

To compare the rate of cell death between MCF-7 and MCF-7/MX, cells were treated with 50 ng/ml TNF-α. We performed phase contrast imaging after 48 hr treatment using an inverted microscope (Labomed, Los Angeles, CA, USA).

***Assessment of the cell viability status by flow cytometry***

A flow cytometry assay was performed for assessment of changing in viability status of MCF-7 and MCF-7/MX cells after 48 hr treatment with TNF-α. 1×10^6^ cells/ml MCF-7/MX cells and MCF-7 cells were cultured in each well of 4-well plates. After overnight incubation, the culture medium of control wells was replaced with fresh medium. The medium of the second wells were exchanged with RPMI 1640 mixed with 50 ng/ml TNF-α. Then plates were put into incubator with 37 ^°^C containing 5% CO_2 _for 48 hr. After incubation, all wells were trypsinized and cells were washed with 1x PBS and suspended in 100 μl of binding buffer containing 5 μl of annexin V-FITC and 5 μl of PI. After 10 min incubation at room temperature in dark, samples were evaluated by a FACS Calibur flow cytometer (BD Biosciences, Heidelberg, Germany).

***Sample preparation for two-dimensional gel electrophoresis (2-DE)***

*Total protein extraction*

Following trypsinization, the harvested cells were suspended in 1 ml cold lysis buffer containing 2 % (w/v) CHAPS, 2 M thiourea, 2 % (w/v) DTT, 6 M urea, ampholyte (pH 3–10) and at the end step protease inhibitor cocktail was added. The cell lysate was sonicated for 60 sec on ice using a probe sonicator (UP 100 H, Germany), retained for 2 hr at 4 ^°^C for protein solubilization and then centrifuged at 10,000 g at 4 ^°^C for 10 min. The pellet was discarded, and protein concentration of supernatant was determined by a Bradford assay. The standard curve was prepared with different concentration of BSA (23). 

***Two-dimensional gel electrophoresis (2D gel electrophoresis)***

In the first step in 2D gel electrophoresis for isoelectric focusing of proteins, the IPG strips were rehydrated in 300 µl of rehydration buffer containing 300 µg protein for 12 hr at 50 V. In the second part the rehydrated IPG strips were laid under different voltages of electrical current sequentially as follows: 250 V for 15 min; 250-8000 V for 150 min; and 8000 V for 11 hr leading to focus proteins at their specific isoelectric point (pI) along the IPG strip. The focused IPG strips were equilibrated by first equilibration buffer containing iodoacetamide for 12 min then were equilibrated by second buffer containing DTT, for 12 min.

In the second dimension of 2D gel electrophoresis, the equilibrated IPG strip was placed on the top of a 12% SDS-poly acrylamide gel (180×180 mm). Electrophoresis was performed at 120 V for 5 hr and then 3 hr at 150 V. Following electrophoresis, gels were stained overnight with colloidal coomassie G250. After the staining step, excess dye was removed and the gels were washed briefly with deionized water (24).

***Analysis of the 2D gel electrophoresis images***

To identify differentially expressed protein spots, the stained polyacrylamide gels were scanned by Image scanner III (Epson, Japan) and the intensity of spots were analyzed with Image Master Platinum 6.0 software (GE Healthcare, USA) which used parametric one to calculate the *P*-value. After normalizing the volume of spots, the density of spots was determined. Spots with more than 1.5 and less than 0.6 fold change and *P*-value<0.05 in MCF-7/MX cell were selected for in-gel digestion and identification by MALDI-TOF/TOF (Matrix Assisted Laser Desorption Ionization-Time of Flight Analyzer) mass spectrometry at the University of York (UK) (25). In analyzing, the spots with scores above 62 were considered as significant MASCOT protein scores.

***Pathway analysis***

Biologic roles of recognized proteins were categorized using PANTHER (Protein ANalysis THrough Evolutionary Relationships) database (http://pantherdb.org) and KEGG PATHWAY Database (https://www.genome.jp/kegg/pathway.html). Protein-protein interaction networks of the selected proteins were predicted by Gene MANIA database (https://genemania.org/) and STRING database (https://string-db.org/).

***Western blotting***

The level of 14-3-3γ and p53 proteins in both MCF-7 and MCF-7/MX cells under treatment with TNF-α were evaluated by Western blotting assay. In brief, cells were lysed followed by adding cold lysis buffer. Following centrifugation pellets were discarded and protein contents of supernatants were evaluated using Bradford protein assay. After SDS-PAGE electrophoresis, proteins were transferred to PVDF (pore size 0.2 µm). Membranes were blocked with 5% skimmed milk for 120 min. The membranes were probed with 1/1000 diluted primary antibodies of interest for 120 min. After several washes, membranes were incubated with horseradish conjugated- secondary antibodies for 90 min. Following addition of ECL, protein bands were visualized (photographed) with ultraviolet illumination gel documentation (UVItec, Cambridge, UK) and analyzed with ultraviolet illumination photo version 99 (UVItec). Based on beta actin band density all protein bands were normalized (26).

***Statistical analysis***

The results were reported as mean±SD. Independent samples T-test were performed to compare means. *P*-values less than 0.05 were considered as significant.

## Results

***Phase contrast imaging***

**Figure 1:**
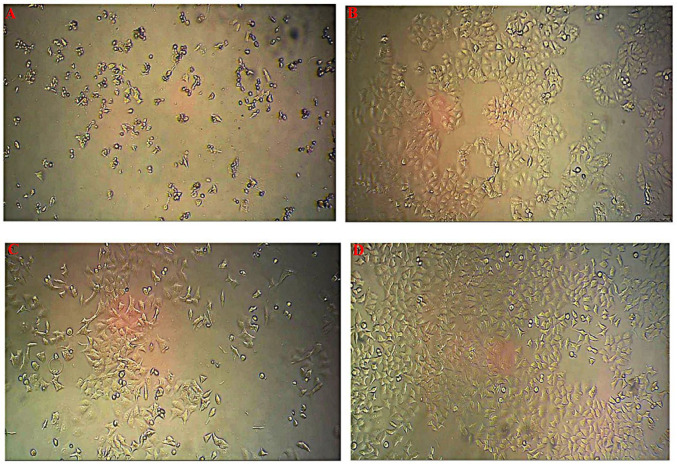
Phase contrast microscope imaging after 48 hr treatment with 50 ng/ml TNF-α

Based on our previous studies TNF-α exerts more cytotoxic effect on MCF-7/MX cells compared to MCF-7 cells. At the first step of this study, we further demonstrate this phenomenon by phase contrast microscope imaging. After 48 hr treatment of both cell lines with 50 ng/ml TNF-α, as shown in Figure 1, the rate of induced cell death by TNF-α in treated MCF-7/MX cells was more than TNF-α-treated MCF-7 cells.

***Assessment of the cell viability status by flow cytometry***

**Figure 2:**
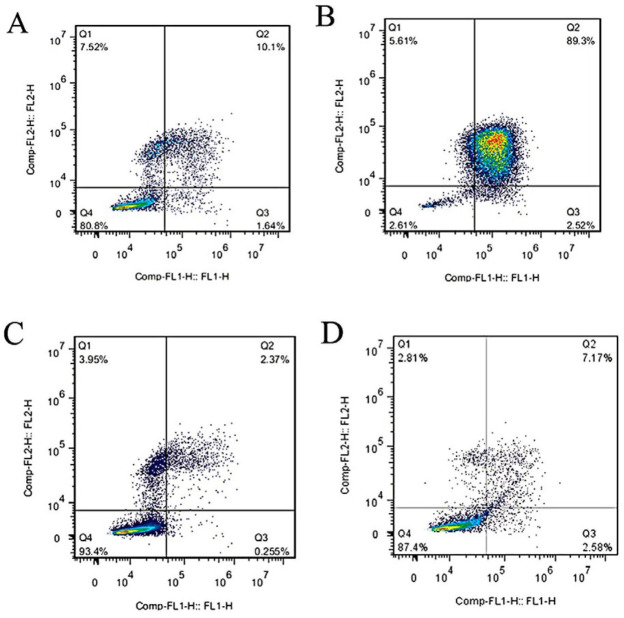
Assessment of the cell viability status by flow cytometry

Cellular viability status of MCF-7/MX and MCF-7 cells exposed to TNF-α for 48 hr were investigated by Annexin V/PI assay. As illustrated in Figure 2, TNF-α treatment increased population of Annexin V+/PI+ cells in MCF-7/MX cells up to 87% demonstrating late apoptosis/necrosis condition. On the other hand, population of TNF-α-treated Annexin V+/PI+ cells were 10.1% representing resistance of *MCF-7* cells against TNF-α induced cell death. 

***Two-dimensional gel electrophoresis***

**Figure 3:**
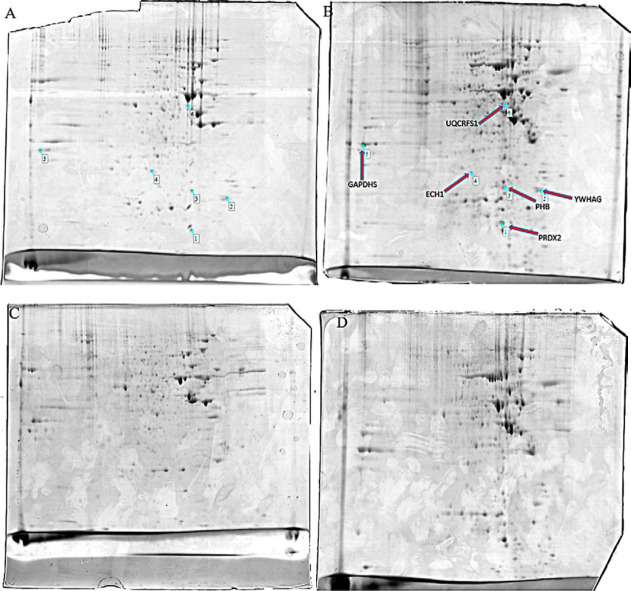
2D gel images. A and C are 2D gels colloidal coomassie blue of TNF-α-treated MCF-7 cells. B and D are 2D gels colloidal coomassie blue of TNF-α-treated MCF-7/MX cells. Six differentially expressed protein spots were identified by MALDI-TOF/TOF and MASCOT software

Proteomes of both TNF-α-treated MCF-7/MX and TNF-α-treated MCF-7 cell lines were compared after 48 hr and 2D gel images are shown in Figure 3. Approximately 40 landmarks per gel were manually selected as primary points for matching by Image Master Software. After matching, as shown in Figure 3, six protein spots indicating fold change more than 1.5 and less than 0.6 were candidates to be identified with mass spectrometry. Characteristics of identified proteins are illustrated in Table 1. 

***Pathway analysis and interaction network***

**Figure 4:**
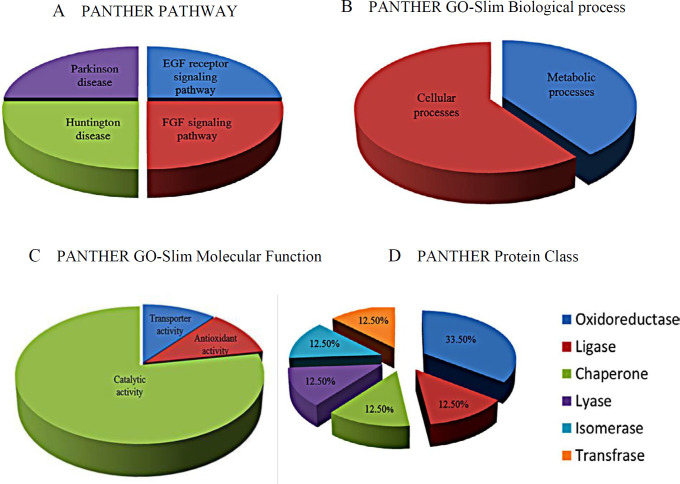
PANTHER classification of six identified proteins

PANTHER classification database was used to indicate biological classification of differentially expressed protein spots. The recognized proteins are related to metabolic pathways involved in FGF signaling pathway, VEGF signaling pathway, EGF receptor signaling pathway, Huntington disease, angiogenesis, p38 MAPK pathway and Parkinson disease (Figure 4). Moreover analyzing of these proteins by KEGG PATHWAY database indicated that the recognized proteins are also involved in different pathways (Table 2) (27).

***Analysis of protein–protein interaction maps of differentiated displayed protein species***

**Figure 5:**
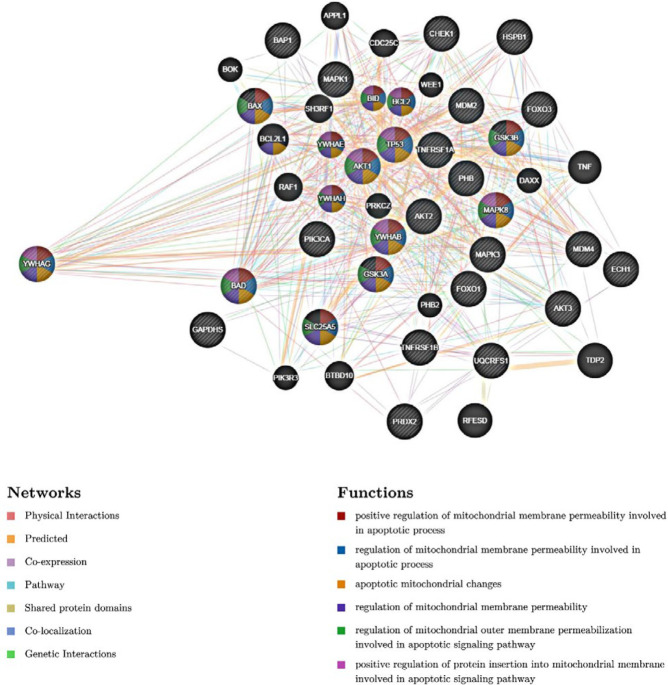
Protein-protein interaction networks between the identified proteins and other proteins. Each protein is identified by a circle. Each color in circle is related to a function

Gene MANIA and STRING databases were applied to explore protein-protein interaction networks between identified protein species and illustrating the pathways being promoted by involvement of these proteins. Gene MANIA database was used for study of physical interaction, function, co-expression, pathways, shared protein domains, co-localization, genetic interactions and prediction of most probable biological events occurring by selected proteins. Analysis of interaction by Gene MANIA database provides a general outlook in interaction of selected proteins between themselves and with other proteins that may be partner with the selected proteins in promoting the pathways that are predicted by Gene MANIA database. As shown in Figure 5, in addition to interactions between the proteins, YWHAG, PRDX2, PHB, ECH1, GAPDHS, UQCRFS1, there are interactions between these proteins and other proteins including BAX, BID, BCL2, AKT1, BAD, TP53, MAPK8 and some other proteins which are mainly related to the regulation of cellular death process. 

**Figure 6:**
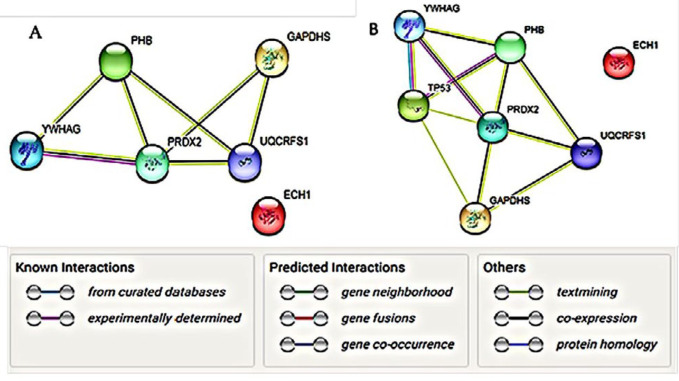
A) Maps of interaction among selected protein species. Nodes and different line colors represent proteins and types of evidence among them respectively. B) Protein–protein interaction maps of selected protein species when p53 was added as a coordinator agent. Interactions between YWHAG, PHB and p53 play important role in the biological pathways including regulated cell death and cell cycle contro

Analysis of interaction map between YWHAG, PRDX2, PHB, ECH1, GAPDHS and UQCRFS1 by STRING database indicated interactions between these proteins (Figure 6A). Based on the main biological process predicted by Gene MANIA database, we decided to study interactions of these six proteins with p53 protein which has fundamental role in triggering and holding of cellular death processes. This is probable that interactions between p53 and six selected proteins is a part of the process triggering cellular death in TNF-α-treated MCF-7/MX and MCF-7 cells (Figure 6B).

***Validation by Western blot analysis***

**Figure 7.**
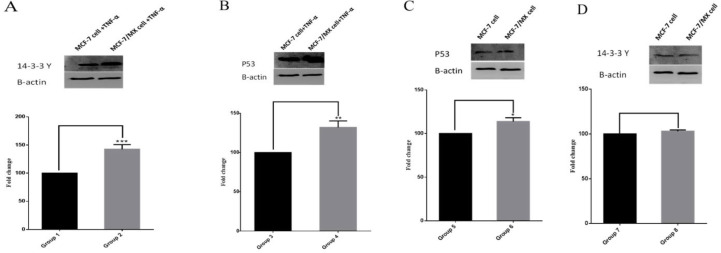
Comparison of 14-3-3γ and p53 proteins expression

Western blot assay was employed for confirmation of the proteomics results for 14-3-3γ protein as one of the recognized differentially expressed proteins in TNF-α-treated MCF-7/MX cells compared to TNF-α-treated MCF-7 cells. Based on the bioinformatics analysis for further elucidation of mechanisms underlying higher vulnerability of MCF-7/MX cells to TNF-α, the expression level of P53 protein was also evaluated by Western blotting. p53 has a crucial connector role in many cellular pathways leading to cell survival, cell arrest and cell death, therefore assessment of p53 expression in both cell lines under TNF-α treatment may be useful in prediction of pathways leading to higher sensitivity of MCF-7/MX cells to TNF-α treatment. As shown on Figure 7, the treatment with 50 ng/ml TNF-α led to upregulation of 14-3-3γ and p53 proteins in MCF-7/MX cells. Intensity analysis of 14-3-3γ and p53 proteins bands indicated that 14-3-3γ and p53 proteins expressed respectively 1.426 and 1.322 folds higher in TNF-α-treated MCF-7/MX cells compared to TNF-α-treated MCF-7 cells. Comparisons of 14-3-3γ and p53 proteins expression between all samples were showed in Figure 7.

## Discussion

Cytokines play crucial roles in the development of immune responses against pathogenic factors such as tumor cells (28). Employing cytokines for developing efficient cancer therapy procedures have been considered for decades. The first applied cytokine for immunotherapy of cancer was TNF-α, but accompany with its ability for inhibition of tumor progression, systemic administration of TNF-α led to severe toxicity (29). To reduce the systemic toxicity, TNF-α has been administrated by delivery systems such as loco regional drug delivery system and tumor targeted delivery (30, 31). Recent findings indicated that TNF-α sensitized breast cancer cells against radiotherapy and chemotherapy (32).

Study of TNF-α effects on multidrug resistant cell lines has indicated that some MDR cell lines such as 8226/D, CEM/V, FLC-DXR and B16-DXR, represented higher vulnerability to cytotoxic effects of TNF-α compared to their drug-sensitive parental cell lines (18, 33). Increased production of TNF-α in MCF-7/ADR (Adriamycin) human breast cancer cell line due to transfection of these cells by TNF-α gene resulted in a significant reversal of resistance to ADR in this cell line. Some compounds have CS ability and can selectively kill MDR cells more than parental cells from which they were derived (34). Based on previous findings about higher cytotoxic effects of TNF-α on some MDR cancer cells compared to sensitive cancer cells, introducing TNF-α as new member of agents with CS ability is plausible (4).

More vulnerability of MCF-7/MX cell line, a mitoxantrone resistant derivative of human breast carcinoma cell line MCF-7, to the cytotoxic effects of TNF-α in comparison with its parental cell line has been reported by our group. Our pervious findings revealed that treatment of MCF-7/MX cells with TNF-α increases ROS level and phosphorylation levels of RIP1 and JNK. On the other hand, using Necrostatin 1, an inhibitor of RIP1 kinase activity and necroptosis, diminished cytotoxic effects of TNF-α in MCF-7/MX cells (21, 22). In this study, we used a proteomics approach to further elucidate possible mechanisms that may be involved in higher cytotoxic effect of TNF-α on treated MCF-7/MX cells.

In this study, phase contrast imaging and Annexin V/PI analysis confirmed our previous results. Significant enhancement of TNF-α-treated MCF-7/MX cell in the Annexin V/PI positive region compared to control and TNF-α-treated MCF-7 cells revealed induction of late apoptosis/necrosis in these cells. 2D gel electrophoresis analysis demonstrated six proteins including PRDX2, YWHAG, PHB, ECH1, GAPDHS, UQCRFS1 with noticeable variation in expression in TNF-α-treated MCF-7/MX cells versus TNF-α-treated MCF-7 cells. These protein species were functionally involved in cellular processes, metabolic pathways, biological regulation processes, immune system processes and localization. Biological functions of the selected proteins according to KEGG pathway are shown in Table 2. Prediction of protein–protein interactions and their probable pathways by STRING and Gene MANIA databases showed that variations in the expression of these proteins specially PRDX2, YWHAG, PHB promoted TNF-α-treated cells toward cellular death pathways.

One of the overexpressed proteins in TNF-α-treated MCF-7/MX was 14-3-3γ protein which belongs to a highly conserved 30 kDa portions family (35). Seven isoforms (β, γ, ε, ζ, η, σ and τ) of this family have been known in mammals (36). The mammalian 14-3-3 proteins have serine/threonine phosphorylation binding sites playing major role in the control of proteins trafficking through some proteins such as Forkhead box protein O (FOXO) (27, 35). The 14-3-3 protein family members have crucial roles in vital cellular processes including metabolism, protein localization, signal transduction, cell death and cell-cycle regulation. The 14-3-3 γ protein is an important member of the 14-3-3 proteins family. It involves in maintaining of cellular homeostasis, signal transduction and cellular protein phosphorylation such as phosphorylation of PHB protein (37, 38).

**Figure 8:**
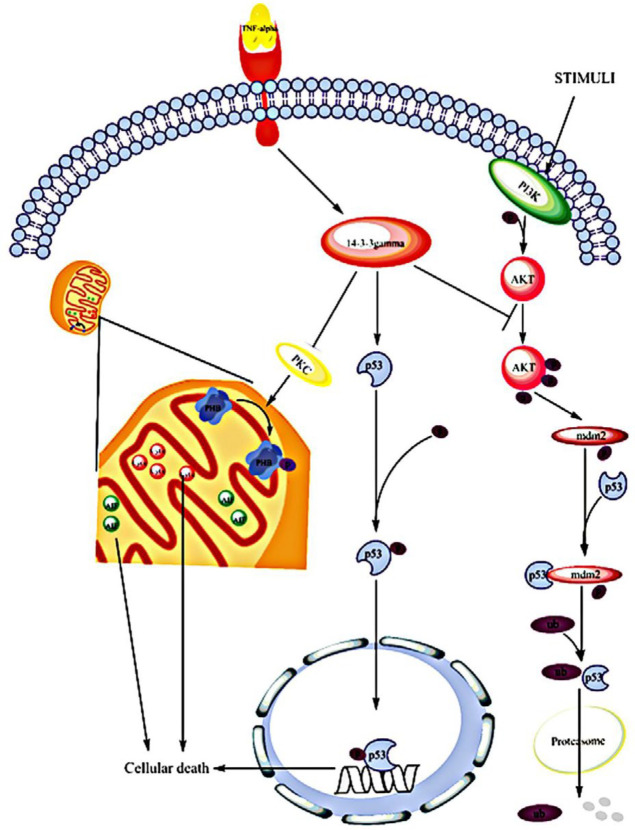
TNF-α signaling pathway based on 14-3-3γ protein function. As mentioned in the text, exposure to TNF-α can lead to overexpression of 14-3-3γ protein in MCF-7/MX cells. This protein interacts with many proteins such as PKC and p53. 14-3-3γ acts as an inhibitor for PKC activity leading to blocking of PHB phosphorylation. Inhibition of PHB phosphorylation elevates mitochondrial permeability and facilitates regulated cell death procedures. 14-3-3γ also elevates stability and activity of p53 via inhibition of AKT phosphorylation which might be another mechanism connecting 14-3-3γ to cytotoxic effects of TNF-α in MCF-7/MX cells

14-3-3γ induced p53 stability and its activity through binding to MDMX mediating p53 proteasomal degradation by ubiquitination (39). MDM2 and its assistant protein, MDMX, have E3 ubiquitin ligase activity. Both proteins have a p53 binding site at the N terminal and RING finger domains at the C terminal. Attachment of MDM2 and MDMX to p53 inhibits its activity (40-42). It has been indicated that MDMX binds to 14-3-3γ more efficiently than other isoforms. Therefore, probably in response to stress elevating p53 expression and activation, 14-3-3γ has a crucial role in the control of cellular death and cell cycle arrest via enhancement of stabilization and activation of p53 (Figure 8) (39). Furthermore, the pro-apoptotic function of another tumor suppressor gene BAP1 that is mostly deleted or mutated in various human cancer types, is facilitated by attachment to 14-3-3 protein (43). Similar to other members of 14-3-3 protein family having binding sites for phosphorylated proteins, 14-3-3γ has a binding site for members of MAPK pathway (such as JNK, p38 and ERK1/2) and PI3K/AKT pathway (such as Foxo, BAD and BAX) (Figure 8) (44).

Upregulation of 14-3-3γ in uterine leiomyoma cells and uterine leiomyoma immortal stem cells significantly elevated cell apoptosis compared to control group and decreased phosphorylated levels of some of the signaling molecules such as p-AKT1, p-AKT2 and p-Foxo1(27, 36). AKT1 and the related AKT2 activation occurs through dual phosphorylation by PI3-kinase (45). The activated form of AKT1 and AKT2 elevate survival ability of cells through phosphorylation and inactivation of some components of the cell death machinery (46). Un-phosphorylated form of FOXO1 is localized to the nucleus and upregulates the expression of some genes involving in programmed cell death such as Fas ligand (FasL) (47, 48). Therefore, reduction of phosphorylation status of p-Akt1, p-Akt2 and Foxo1 following to overexpression of 14-3-3γ can down regulate AKT dependent survival pathways and upregulate cellular death pathways through decreasing of Foxo1 phosphorylation. 

We have demonstrated that TNF-α treatment leads to enhancement of phosphorylation of Akt at Ser473 in MCF-7 cells compared to MCF-7/MX cells. Enforced activation of PI3K signaling and subsequently enhancement of Akt phosphorylation in MCF-7 cells attenuated cytotoxic effects of TNF-α. On the other hand, inhibition of PI3K signaling by a chemical inhibitor sensitized MCF-7 cells to cytotoxic effects of TNF-α demonstrating pivotal role of Akt phosphorylation in response of *MCF-7* and *MCF-7/MX* cells to TNF-α treatment (22). Outcome of the present study indicated that 14-3-3γ expression level was 1.4 folds higher in TNF-α-treated MCF-7/MX cells compared to TNF-α-treated *MCF-7* cells. As mentioned above, 14-3-3γ induces cell death via decrease in the phosphorylation of some of signaling molecules such as p-Akt1, p-Akt2, and p-Foxo1. Therefore, it is plausible that overexpression of 14-3-3γ in treated MCF-7/MX cells is involved in the reduced Akt phosphorylation and elevated vulnerability of these cells to cytotoxic effects of TNF-α. Phosphorylation of transcription factor Foxo1 by Akt leads to its translocation from the nucleus and degradation by proteasome causing inhibition of transcription of genes involved in regulated cell death (47). Investigating direct role of 14-3-3γ in the phosphorylation status of Akt in TNF-α-treated *MCF-7* and MCF-7/MX cells as well as implication of this pathway in collateral sensitivity are open to question in future studies.

In addition to 14-3-3γ higher expression, western blot analysis showed overexpression of p53 protein in TNF-α-treated MCF-7/MX cells compared to TNF-α-treated MCF-7 cells. Activation and stabilization of tumor suppressor protein p53 by 14-3-3γ protein have been reported (39), therefore, it is probable that overexpression of p53 under this condition is due to increased expression of 14-3-3γ protein. Some pathways that are relevant to 14-3-3γ function have been shown in Figure 5, each color is related to a function and multi-colored proteins such as 14-3-3γ and p53 are mainly involved in pathways leading to cellular death. p53 is involved in the regulated cell death pathways including apoptosis and necroptosis. Various studies have demonstrated role of p53 in activation of cathepsin Q and subsequently induction of ROS mediated necroptosis (49-51). A physical interaction between p53 and mitochondrial permeability transition pore (PTP) regulator, cyclophilin D (CypD), was also reported. Under oxidative stresses the p53 protein was accumulated in matrix of mitochondria and induced necrosis through PTP opening via interaction with CypD (52). In another study p53 depletion led to impairment of ROS induced necrotic cell death in mouse embryonic fibroblasts, human colorectal and human breast cancer cell lines (53). It has been reported that the p53 protein may also has a noticeable role in triggering RIPK1 kinase activity and RIPK-induced necroptosis. Activation of p53 upregulates a long-noncoding RNA called necrosis-related factor. On the other hand, miRNA-873, which suppresses expression of RIPK1/RIPK3, introduced as target for necrosis-related factor. Therefore, induction of p53 can upregulate RIK1/RIPK3 by upregulation of necrosis-related factor and subsequently downregulation of miRNA-873 (54). Since our previous studies suggested a role for RIP1 and ROS in TNF-α induced non-apoptotic cellular death in MCF-7/MX cells, it can be concluded that overexpressed p53 is involved in this phenomenon by induction of ROS and upregulation of RIPK1/RIPK3.

Prohibitins are a highly conserved group of proteins that are predominantly located in the mitochondria, nucleus, and the plasma membrane (55). PHB has a main role in many cellular pathways that some of them were shown in Table 2. Silencing PHB in MCF-7 cells increased the ratio of some survival features including proliferation, cell distribution in S phase and decreased cell count in the G1–G0 phase of the cell cycle (56). 14-3-3γ protein inhibits phosphorylation of PHB through inhibition of PKC-δ activation and increased cell death (Figure 8) (57). It has been shown that in the presence of apoptotic signals in the human breast carcinoma cell line T47D under treatment with camptothecin, both PHB and p53 translocate from the nucleus to the cytoplasm, and this demonstrated the correlation of PHB and p53 with the onset of apoptosis (58). Blocking of PHB phosphorylation leads to elevation of mitochondrial permeability facilitating mitochondrial dependent cell death (57). Based on these results, we suggest that upregulated expression of 14-3-3γ in TNF-α-treated cells in addition to supportive effect on stability and activity of p53 may destabilize mitochondria through inhibition of PHB phosphorylation facilitating cellular death. 

In spite of overexpression of 14-3-3γ, PHB and P53 proteins in TNF-α-treated MCF-7/MX cells compared to TNF-α-treated MCF-7 cells, our study indicated that under TNF-α treatment the expression of PRDX2 protein decreased in treated MCF-7/MX cells with TNF-α. PRDX2 plays crucial antioxidant role specially scavenging H_2_O_2_ in the cells and its expression is dependent on levels of ROS production. Our previous results indicated that following TNF-α treatment ROS levels in MCF-7/MX cells is increased noticeably compared to *MCF-7* cells. Decreasing of the PRDX2 protein level under treatment of MCF-7/MX cells with TNF-α can be one of the main factors leading to more susceptibility of TNF-α-treated MCF-7/MX cells to elevation of ROS production compared to TNF-α-treated MCF-7 cells. In our previous studies catalase and superoxide dismutase activities were also significantly impaired in MCF-7/MX cells treated with TNF-α compared to their parental cells. On the other hand scavenging intracellular ROS after TNF-treatment using antioxidant agents in MCF-7/MX cells decreased cytotoxic effects of TNF-α in MCF-7/MX cells demonstrating pivotal role of ROS in the TNF-α mediated cellular death (59). Taken together, it seems impaired antioxidant capacity of the resistant cell line is involved in the accumulation of ROS and induction of regulated cell death pathways by TNF-α in these cells. 

A) Treated *MCF-7/MX* cells with TNF-α for 48 hr. B) *MCF-7/MX* cells without treatment. C) Treated *MCF-7* cells with TNF-α for 48 hr. D) *MCF-7* cells without treatment

A) Treated MCF-7 cells with TNF-α. B) Treated MCF-7/MX with TNF-α. C) MCF-7 cells without treatment. D) MCF-7/MX cells without treatment. TNF-α-treated MCF-7/MX cells were 5.61 % Annexin V-/PI+(Q1), 89.3 % Annexin V+/PI+ (Q2), 2.52 % Annexin V+/PI-(Q3), and 2.61% Annexin V-/PI-(Q4) whereas TNF-αtreated-MCF-7 cells showed 7.52 % Q1, 10.1 % Q2, 1.64 % Q3 and 80.8 % Q4

**Table 1:** Differentially expressed protein species in TNF-α-treated MCF-7/MX cells and TNF-α-treated MCF-7 cells which identified by MALDI-TOF/TOF and MASCOT software

Protein Name /Gene name	Theoretical MW (kDa)	Calculated pI	Fold-change (TNF-α-treated *MCF-7*/*MX* cells and TNF-α-treated *MCF-7* cells)	Accession number	Protein Score	% Sequence coverage	Expectation value	Matched peptide number	Matched peptide sequence
PRDX2 (Peroxiredoxin 2)	22049	5.66	0.287	P32119	347	6.57%	7.6e-05	6	K.TDEGIAYR.G R.IGKPAPDFK.A K.ATAVVDGAFK.E R.LSEDYGVLK.T R.RLSEDYGVLK.T R.QITVNDLPVGR.S
YWHAG (14 3-3 ɣ)	28456	4.80	2.602	P61981	122	6.47%	0.016	2	K.DSTLIMQLLR.D K.NVTELNEPLSNEER.N
PHB (Prohibitin)	29843	5.57	0.407	P35232	683	9.55%	0.0014	8	K.QVAQQEAER.A R.FDAGELITQR.E K.DLQNVNITLR.I R.ILFRPVASQLPR.I R.IFTSIGEDYDER.V R.KLEAAEDIAYQLSR.S K.AAELIANSLATAGDGLIELR.K K.FGLALAVAGGVVNSALYNVDAGHR.A
ECH1(Delta (3,5)-Delta (2,4)-dienoyl-CoA isomerases, mitochondrial)	35,816	6	1.830	Q13011	299	5.18%	2.3e-05	4	R.YQETFNVIER.C R.YCAQDAFFQVK.E K.MMADEALGSGLVSR.V K.EVDVGLAADVGTLQR.L
GAPDHS (glyceraldehyde-3-phosphate dehydrogenase)	36201	8.57	1.791	P04406	490	7.76%	3.8e-05	6	K.AGAHLQGGAK.R R.GALQNIIPASTGAAK.A R.VPTANVSVVDLTCR.L K.LVINGNPITIFQER.D K.LVINGNPITIFQERDPSK.I K.VIHDNFGIVEGLMTTVHAITATQK.T
UQCRFS1(Cytochrome b-c1 complex subunit Rieske, mitochondrial)	53297	6.44	3.042	P31930	677	5.2%	0.00089	9	K.NRPGSALEK.E R.IAEVDASVVR.E R.RIPLAEWESR.I R.LCTSATESEVAR.G R.ADLTEYLSTHYK.A K.EVESMGAHLNAYSTR.E R.NALVSHLDGTTPVCEDIGR.S R.VASEQSSQPTCTVGVWIDVGSR.F K.YIYDQCPAVAGYGPIEQLPDYNR.I

*In analyzing of the 2D images spots with scores above 62 were considered as significant MASCOT protein score

**Table 2:** Biological functions of differentially expressed proteins according to KEGG pathway

Protein Name /gene name	Biological function according to KEGG pathway
PRDX2 (Peroxiredoxin 2)	Acting on a peroxide as acceptor, peroxidases
YWHAG (14 3-3 ɣ)	Hippo signaling pathway, cell cycle, oocyte meiosis, PI3K-Akt signaling pathway, Epstein-Barr virus infection, viral carcinogenesis
PHB (Prohibitin)	Signaling and cellular processes, mitochondrial biogenesis
ECH1(Delta (3,5)-Delta (2,4)-dienoyl-Co-A isomerases, mitochondrial)	A part of the Peroxisome
GAPDHS (glyceraldehyde-3-phosphate) dehydrogenase)	Glycolysis/gluconeogenesis, metabolic pathways, biosynthesis of secondary metabolites
UQCRFS1(Cytochrome b-c1 complex subunit Rieske, mitochondrial)	Metabolic pathways, oxidative phosphorylation, two-component system, cardiac muscle contraction, thermogenesis, non-alcoholic fatty liver disease (NAFLD), Alzheimer's disease, Parkinson's disease, Huntington's disease

A) Group 1: TNF-α-treated *MCF-7* cells (14-3-3 γ protein expression), Group 2: TNF-α-treated *MCF-7/MX* cells (14-3-3 γ protein expression). B) Group 3: TNF-α-treated MCF-7 cells (p53 protein expression), Group 4: TNF-α-treated MCF-7/MX cells (p53 protein expression). C) Group 5: Untreated MCF-7 cells as negative control (p53 protein expression), Group 6: Untreated MCF-7/MX cells as negative control (p53 protein expression). D) Group 7: Untreated MCF-7 cells as negative control (14-3-3 γ protein expression), Group 8: Untreated MCF-7/MX cells as negative control (14-3-3γ protein expression). The data indicate the mean±SD (n=3). *P<0.05, **P<0.01 and ***P<0.001 significantly different from corresponding control

## Conclusion

Results of this study indicate that under TNF-α treatment 14-3-3γ, p53 and PRDX2 proteins were differentially expressed in MCF-7/MX cells compared to *MCF-7* cells. Upregulation of 14-3-3γ and p53 and down regulation of PRDX2 can trigger the programmed cellular death processes including apoptosis, necroptosis and other cellular death mechanisms. Since our previous studies demonstrates caspases are not involved in higher cytotoxic effects of TNF-α in MCF-7/MX cells and emphasized the role of RIPK1 and ROS in this process, it seems non-apoptotic cellular death mainly necroptosis is the main mechanism underlying in this phenomenon. More studies on TNF-α and other compounds with CS ability and their therapeutic effects on MDR cancer cells can lead to finding new targets and therapeutic procedures to overcome resistance in MDR cancer cells. 
